# Everyday disease diplomacy: an ethnographic study of diabetes self-care in Vietnam

**DOI:** 10.1186/s12889-022-13157-1

**Published:** 2022-04-25

**Authors:** Tine M. Gammeltoft, Thị Huyền Diệu Bùi, Thị Kim Dung Vũ, Đức Anh Vũ, Thị Ái Nguyễn, Minh Hiếu Lê

**Affiliations:** 1 Department of Anthropology, University of Copenhagen, Øster Farimagsgade 5, DK-1353 Copenhagen K, Denmark; 2 Thai Binh University of Medicine and Pharmacy, 373 Ly Bon Street, Thai Binh Thai Binh City, Vietnam

**Keywords:** Chronic conditions, Diabetes, Everyday life work, Self-care, Vietnam

## Abstract

**Background:**

Understanding people’s subjective experiences of everyday lives with chronic health conditions such as diabetes is important for appropriate healthcare provisioning and successful self-care. This study explored how individuals with type 2 diabetes in northern Vietnam handle the everyday life work that their disease entails.

**Methods:**

Detailed ethnographic data from 27 extended case studies conducted in northern Vietnam’s Thái Bình province in 2018–2020 were analyzed.

**Results:**

The research showed that living with type 2 diabetes in this rural area of Vietnam involves comprehensive everyday life work. This work often includes efforts to downplay the significance of the disease in the attempt to stay mentally balanced and ensure social integration in family and community. Individuals with diabetes balance between disease attentiveness, keeping the disease in focus, and disease discretion, keeping the disease out of focus, mentally and socially. To capture this socio-emotional balancing act, we propose the term “everyday disease diplomacy.” We show how people’s efforts to exercise careful everyday disease diplomacy poses challenges to disease management.

**Conclusions:**

In northern Vietnam, type 2 diabetes demands daily labour, as people strive to enact appropriate self-care while also seeking to maintain stable social connections to family and community. Health care interventions aiming to enhance diabetes care should therefore combine efforts to improve people’s technical diabetes self-care skills with attention to the lived significance of stable family and community belonging.

## Background

Across the world, low- and middle-income countries (LMIC) are currently seeing a rapid increase in non-communicable diseases (NCDs), including diabetes. Although rising diabetes rates is a globally shared predicament, the rise is more marked and the consequences more severe in socio-economically disadvantaged parts of the world [1–5]. Millions of people in resource-constrained settings are currently living with complications and reduced well-being due to diabetes, posing obstacles to progress towards achieving SDG3 (healthy lives and well-being for all) [2, 4]. In this context, global health actors such as the World Health Organization (WHO) currently draw attention to the importance of self-care for SDG achievement, noting, in the words of the WHO, that “the provider-to-receiver model that is at the heart of many health systems must be complemented with a self-care model through which people can be empowered to prevent, test for and treat disease themselves” [6].

In LMIC, self-care for chronic disease often comes on top of existing burdens of poverty, inequality, and infectious disease, adding new forms of stress to lives that are already lived under pressure [7, 8]. Further, while clinical guidelines emphasize the individual practices that self-management of a disease such as diabetes entails, previous research has pointed to the vital roles played by others – such as family members and peers – in daily disease management [9–13]. Although the importance of informal support persons in self-care for health is often recognized in theory [6], there is a lack of empirical knowledge of the ways in which NCD self-care is embedded in everyday family- and community lives in the global South and of the social life challenges that self-care entails in these settings [14].

In Vietnam, a lower middle-income country, at least 6% of the population has been diagnosed with type 2 diabetes (T2D) and many are assumed to live with pre- or undiagnosed diabetes [15, 16]. Given that diabetes affects not only the diagnosed person, but also family- and household members, a considerable proportion of Vietnam’s population of 98 million people are currently living under the impact of this chronic disease. Along with other NCDs, diabetes poses significant threats to the country’s continued economic development, and the Vietnamese government is making concerted efforts to address the growing NCD burden through enhanced prevention and management [17]. To date, however, hardly any qualitative research has been conducted in Vietnam on the perceptions and experiences of people diagnosed with T2D or their social supporters. On this background, this study aimed to explore the everyday impact of T2D (hereafter, “diabetes”), highlighting particularly the social contexts of everyday disease self-care.

In this paper, we attend to the everyday life work that diabetes self-care entails. We take the term “everyday life work” from Juliet Corbin and Anselm Strauss’s classic research on the management of chronic illness at home [18, 19]. Corbin and Strauss define the work of chronic illness management as “a set of tasks performed by an individual or a couple, alone or in conjunction with others, to carry out a plan of action designed to manage one or more aspects of the illness and the lives of ill people and their partners” [19]. We find the concept of *work* useful, as it draws attention to the everyday labour that chronic disease demands, for patients as well as their supporters, while also helping to throw analytical light on the wider social settings – families, neighbourhoods, communities – in which everyday disease self-care unfolds [20].

## Methods

This study was conducted in Vietnam’s Thái Bình province by a Vietnamese-Danish research team under the auspices of the interdisciplinary and collaborative research project VALID (“Living Together with Chronic Disease: Informal Support for Diabetes Management in Vietnam”). Thái Bình province is located in the Red River Delta, a densely populated rice-producing area. In many respects, the diabetes situation in Thái Bình reflects the national situation in Vietnam: firstly, the prevalence of diabetes is around 6% as in the nation as a whole; secondly, health care strategies aim to shift NCD management from secondary/tertiary levels of care to the primary level; and thirdly, this planned shift to primary level care is still underway, and people with diabetes currently continue to obtain care at provincial hospitals. In Thái Bình, as elsewhere in Vietnam, hospital overcrowding places constraints on health care delivery, resulting in long waiting times, high costs, and strained patient-provider relations [21–23].

This ethnographic research was conducted as an extended case study involving 27 individuals with type 2 diabetes and their households^1^. The extended case study was selected as the primary research method due to its capacity to generate rich ethnographic data through research conducted over time [24]. Participants were recruited in a rural commune of Thái Bình’s Vũ Thư district through convenience sampling among people with type 2 diabetes living in the uptake area of the commune health station. The participants were approached face-to-face by local health care workers and invited to take part in the study. Fifteen individuals were enrolled in November 2018 and 12 in April 2019^2^. The sample included 14 women and 13 men, aged 67.1 years on average. Of the 27 individuals, two were living from social support from the state, 13 had retired and received a pension, eight had retired and lived from support provided by their families, and four were working. Two lived alone, while eight shared a household with their spouse, and 17 lived in two- or three-generation families, most often together with an adult son and his family (see Table 1: Descriptive characteristics of study participants at the time of recruitment). To protect anonymity, all participant names are pseudonyms.

**Table 1:** Descriptive characteristics of study participants at the time of recruitment (2018/2019)

Characteristics		Participants *(N* = 27)	% of total
Age	Mean (range)	67.1 (49–81)	
Occupation/income	Service/farming	4	14.8
	Retired, with pension	13	48.1
	Retired, with family support	8	29.6
	Receiving social assistance	2	7.4
Self-reported years with diabetes	Mean (range)	8.1 (1–29)	
Gender	Female	14	51.9
	Male	13	48.1
Marriage status	Married	22	81.4
	Widowed	4	14.8
	Divorced	1	3.7
Household	Living alone	2	7.4
	Living with spouse (one generation)	8	29.6
	Living in two-generational household	6	22.2
	Living in three-generational household	11	40.7
Medicine	Oral medication	18	66.6
	Insulin	9	33.3

Data collection was undertaken between November 2018 and May 2020. Each initial case visit included a semi-structured interview which was conducted in the home of the person with diabetes, exploring experiences of daily life with the disease. When desired by the participants, family members were present during home visits. The initial interviews were followed up by informal visits, conversations, and participant observation during diabetes check-up visits at the hospital. The research team met with one participant once; twelve participants twice; and fourteen participants three times or more. During follow-up visits, the researchers worked with an open ethnographic approach, shadowing the participants and engaging in informal conversations with them [25]. All interviews and conversations were conducted in Vietnamese, voice-recorded, and transcribed verbatim, and detailed fieldnotes were taken. Interview transcripts and fieldnotes were coded by the authors, with codes developed from the original research questions and the researchers’ fieldnotes. Main categories in the coding system were life history; family situation; work; domestic economy; diabetes history; understandings of the disease; daily disease management (subthemes: diet, alcohol, exercise); experiences with hypo/hyperglycaemia; diabetes check-ups/interactions with the health care system; medications; complications; other health problems; emotional life; sexual life; support from family and community; and relations to other people with diabetes in the community. The analysis was performed on the Vietnamese language transcripts and only the quotes used for this article were translated into English. Transcripts and codes were not returned to participants for comment or feedback.

## Results

“Since then I have lived with the floods” (“từ đó cứ sống chung với lũ vậy”). When describing what their lives were like after the diabetes diagnosis, people repeated this expression again and again. For the inhabitants of this Red River Delta area, the idiom “living with the floods” brings to mind nature’s overwhelming forces: bursting dikes, rice fields turned into oceans, loss of livestock and lives. When confronted with such powerful forces, the best survival strategy is to “live with the floods,” accepting what comes. People cited this old adage, with its resonances of accommodation and acceptance, to convey their submission to the diagnosis they had received. Such submission did not, however, entail passivity. On the contrary, all research participants made concerted efforts to manage their diabetes in the best possible way. Diabetes was a plight that demanded daily work: the work of diet management, exercise, intake of medicine, and health care check-ups. Our analysis of the ethnographic material pointed to four main dimensions of such everyday life work: efforts to regulate blood sugar; to regulate medicine; to regulate food and drink; and to regulate emotions. Four ethnographic vignettes drawn from the extended case study serve to illustrate how this everyday life work unfolded in the context of participants’ daily lives:

### Regulating blood sugar: the work of dual attention

Dương was born in 1948. During the Second Indochina War, he fought in the North Vietnamese Army, spending considerable time in Vietnam’s Central Highlands where U.S. forces spread the highly toxic herbicide dioxin Agent Orange. When asked if he felt worried when he was diagnosed with diabetes, he promptly responded, “No! The war taught me to live between life and death. I’m used to that.” Actually, Dương said, he feels lucky. He came back from the war alive. Many did not. Therefore, when he was diagnosed with diabetes three years ago, he did not feel it was a big problem, except that now he must live “according to the doctors’ regimen” (“theo phác đồ của bác sĩ”). Since he was diagnosed, he has changed his diet, eating less white rice, less meat, and fewer sweet fruits. He has attended his monthly diabetes check-ups meticulously, taken his medicine, and exercised every day, using a bike that he bought when he was diagnosed. He makes his own tea from guava leaves which are said to be effective against diabetes, and in between meals he drinks a home-made concoction based on roasted and grinded lotus seeds, green beans, and peanuts dissolved in water. “With this disease,” he says, “it is oneself who decides whether or not blood sugars are stable.”

Despite all these efforts, keeping blood sugars balanced is not easy for Dương. His blood sugars are measured only once a month when he goes for his routine check-up at the hospital. The rest of the time he manages by attending carefully to his own body. Often, he feels suddenly tired, uncomfortable and dizzy, sweat trickling, arms and legs trembling. Sometimes he has trouble standing upright, black spots dancing in front of his eyes. In these situations, he sits down for a while until he feels stable again. Sometimes, when feeling unwell, he drinks his home-made concoction or eats instant noodles or sugar-free biscuits. During our conversation, it becomes clear that Dương has never received information from health care providers about hypoglycaemia or how to handle it. Instead, he has learnt through experience how to handle sudden feelings of unease. But as he goes about his daily life, he tries not to think too much about the feelings of exhaustion and weakness that sometimes overwhelm him. Focusing too much on one’s disease, he holds, will only aggravate it. “It’s important that one’s thoughts are relaxed and serene,” he emphasizes, speaking in an authoritative tone. “Living with diabetes, one should not attend too much to the disease. Avoiding thinking about it will make it less grave. One must take things as they come, expelling one’s thoughts, not letting them linger.”

For Dương, as for other participants in this study, maintaining stable blood sugars demanded daily work. Since only three of the 27 individuals had a glucometer at home, the majority relied on monthly blood sugar measurements carried out at the hospital in combination with careful attention to the states of their bodies. If blood sugars fluctuated, many said, they would feel weak, tired, tense, and dizzy, arms and legs trembling, sweat trickling. Several research participants had not been informed by health care providers about hypo- and hyperglycaemia, and not all were familiar with these terms. Instead of relying on professional advice, they had developed their own techniques for preventing and handling feelings of physical unease, taking small meals when these sensations arose. Besides this direct work of bodily management, diabetes self-care also entailed more indirect work: while attending closely to their bodies, trying to prevent blood sugar imbalance, many also strove hard *not* to attend to their diabetes, believing like Dương that a mental focus on the disease would only aggravate it. People’s state of being was, in other words, experientially split, as they strove to disregard their disease at the same time as they attended carefully to the states of their bodies. Daily diabetes self-care involved, in other words, a keen attentiveness to one’s body, combined with efforts not to attend to one’s disease – a balancing act that we term “everyday disease diplomacy.”

### Regulating medicines: the work of medication speculation

In the summer of 2012, Ly suddenly started to lose weight. In one month, she lost four kilos. The next month, she lost another four kilos. She was 63 years old at the time and felt puzzled by this sudden weight loss. When she went for a health check, the doctor told her she had diabetes. Hearing this, she felt frightened. She immediately thought of one of her neighbours who had recently died from her diabetes. When she was younger, this woman had lived from doing manual contract labour. Now, in her old age, she could not work anymore, and having no pension, she depended entirely on her children. However, her children did not help her much, and she developed frightening complications, including sores on her feet and bones sticking out of her legs. “With this disease,” Ly says, placing emphasis on her words, “one needs help from the state. Without such help, poor people will suffer. At least I have my pension, but from where can poor people find the money to buy efficient medicine?” When she was younger, Ly worked as a teacher and her husband served in the army. This has given them modest monthly pensions from the state, enough to survive on. They share a household with their eldest son and his family, but financially they live independently. Each month, they spend around a third of their money on medicines for Ly. Besides her diabetes medicine, she also takes medicine to strengthen her heart, her liver, her lungs, and her nervous system.

As she sits in her wooden sofa, wearing a purple floral pyjamas, Ly looks fragile. Since her initial weight loss, she has not been able to put on weight again, and today she weighs only 35 kilos. Still, fearing complications from her diabetes, she eats very little. At night, she often lies wide awake, worrying. She thinks about her medicines. Like most other villagers, she has a health insurance, but this insurance covers only a limited number of medicines – and these medicines, she has found, are not effective. Even though she takes her medicine exactly as prescribed, her blood sugar levels remain much too high. She therefore sometimes goes to the local pharmacist and buys the French medicine *Diamicrone* which many people with diabetes in her area are using. This medicine is much more effective, she says. But it is expensive, and she cannot always afford it. She wishes that the state would ensure that citizens have access to efficient diabetes medicines: “Medicines must be effective. Taking medicines with no effect makes one feel depressed, when one’s blood sugar doesn’t go down, one feels depressed. It’s a problem for people who do not have a lot of money.” Even though Ly struggles with doubts regarding her medications, she does not share these concerns with her children. “When they ask how I am,” she says, “I just say that I’m fine. I don’t want to burden them. I’m still in good health, I can still work. They don’t need to take care of me yet, not until I get old and weak.”

For many research participants, diabetes medications were a matter of work. Firstly, remembering to take the medications every day required work. Secondly, for those using insulin, there was work involved in giving oneself injections, or asking others to help. But for many, the main work burden associated with diabetes medicines was the work of what we term “medication speculation”: many research participants made constant and concerted efforts to evaluate the effectiveness of their medicines, pondering whether other medicines might work better. Struggling with continuing unbalanced blood sugars and feelings of weakness and unease, many doubted the efficiency of their medicines. Having limited access to biomedical information, due to hospital overcrowding and a lack of counselling, they sought alternative avenues of information, learning from other diabetes patients, studying the Internet, or simply trying out different kinds of medication purchased from local pharmacists or herbal drug sellers. Many also adjusted their medicines themselves, taking larger or smaller doses than prescribed. Undertaking these efforts of medication speculation, people tried not to cause offense to anyone, including the health care providers in charge of their care. While struggling with unresolved doubts and questions, many deliberately avoided asking health care providers for advice, fearing being “scolded” or in other ways reproached. Many also sympathised with health care workers who were obviously overburdened by the number of patients in their consultation, or they feared taking other patients’ time. Further, since they did not want to burden their children, most individuals went about this search for alternative medications with discretion, trying to find their own way in the pharmacy market. Longing for a normal life without disease, some skipped their medications for a period of time, hoping that they could do without them. At issue, again, was everyday disease diplomacy: a careful balancing between disease attentiveness and discretion, as people strove to manage their diabetes without attracting attention or burdening others.

### Regulating food and drink: the work of social integration

“My life now is completely different from what it was before,” Vân says, shrugging his shoulders. Vân is a 56-year-old farmer who was diagnosed with diabetes when he was 54. His 80-year-old father has had diabetes for 22 years, so Vân knows that this is a serious disease: his father’s eyesight has become very poor, and his feet are constantly swollen. The old man lives just around the corner from Vân, sharing a house with Vân’s younger brother and his family. The two families help each other caring for him. The nights are Vân’s responsibility: every evening, he goes to sleep in his father’s room, so that he can help him to go out when he needs to pee, usually around ten times every night. In the morning, Vân returns home, has breakfast with his family, and drives his two grandchildren to school. Seeing his father’s trouble with his diabetes, Vân fears developing complications too. To prevent this, he tries to live according to the guidelines that health providers have given him. He takes a 30-minute walk every morning and sticks to a diabetes-friendly diet, avoiding sweet drinks and fruits, eating very little rice, and avoiding fat meats. His wife is a housewife and very dedicated to their family, Vân says. She usually cooks a range of different dishes, so family meals are not difficult to align with Vân’s diabetes: from the variety of different dishes on the tray, he simply chooses those that are best for him. “I eat a lot of vegetables,” he says, “and tofu”.

But going out with his friends is a different matter. He cannot eat like them anymore, and he feels that his presence takes away the happy, joyful spirit in their group. The “male” foods and drinks that he used to share with his friends include fat meats, dog meat, and lots of rice wine. “Due to my disease,” Vân explains, “I cannot hang out with my friends like I could before. My life has become very different. Before I would eat dog meat and drink rice wine constantly, but now I’ve stopped. I still like the food that I used to eat, but I don’t dare eat it anymore. My friends still come and invite me to go out with them, but I can’t. I joke with them, telling them: ‘At some meals, I eat vegetables, at some meals I eat tofu, at some meals I eat fish. I don’t eat meat anymore. I don’t eat pig’s blood pudding anymore.’ My friends used to come every day to invite me to go out to eat pig’s blood pudding. Now I don’t go anymore. And when there are celebrations in our village, I ask my wife to attend on my behalf.”

Among the most demanding tasks that research participants undertook in connection with their diabetes was the work of changing their habits of eating and drinking. After their diabetes diagnosis, most adjusted their diets, reducing their intake of the foods that health care providers warned them against – white rice, sweet fruits, and fat meats – while increasing their intake of foods considered compatible with their diabetes, such as boiled vegetables and non-sugary fruits such as guava. These dietary changes had far-ranging – and highly gendered – social implications. Women would often express deep concerns about the impact of their dietary changes on their families, fearing that the cosy family atmosphere at meals would be altered if others were too conscious of their disease. In some cases, people with diabetes would continue to eat the same diet they had always eaten, out of concern that dietary changes would affect their families negatively. Men, in contrast, would often express concern about the impact of their diabetes on their abilities to socialize with their friends, since male sociality in northern Vietnam often involves alcohol and “fat” meats such as dogmeat, and many described being subjected to peer pressure to continue drinking alcohol despite their disease [27]. Since commensality – the joyous eating and drinking together – is at the heart of daily lives in rural Vietnam, both women and men engaged in active disease diplomacy when it came to their diets and meals, trying to minimize the impact of their disease on others. Women most often did so with a focus on the domestic arena, while men enacted dietary/drinking disease diplomacy in the public realm when socializing with other men.

### Regulating emotions: the work of social discretion

“What I need most is health” (“*cần nhất là cái sức khỏe”*), Bích says. Her voice is hopeful. “I’m getting old and weak, but I so much want to reduce this disease.” Bích was diagnosed with diabetes seven years ago, at the age of 58. She found out that she had diabetes because her entire body started to tremble. She went to the local health station where staff brought her to the provincial hospital’s emergency room. Here, doctors found that her blood sugar count was 23. Now it is usually around 8. Her eyesight is deteriorating for each day, she says, and she has lost feeling in her feet, so she walks with difficulty. Bích receives us in a beautiful building with intricately carved window ornaments and pillars with classical script, its red tile roof glittering in the autumn sun. This building, she says, belongs to her husband’s lineage. Due to the couple’s poverty, the lineage head has given Bích and her husband permission to live here. “Sometimes we have no money for food,” Bích says, shrugging her shoulders. “But having diabetes, I’m not allowed to eat anything anyway. And my husband is often too weak to eat much.” Her husband has been ill for years, struggling with frequent seizures, and Bích’s modest earnings from farming and small trade are their main source of income. Their son lives and works in another province, and their daughter-in-law works long hours in a nearby factory. It is Bích who takes care of their granddaughters, aged two and four, when their mother is at the factory. Sharing the work with her husband’s siblings, she also takes care of her husband’s elderly parents.

Bích feels intensely worried about her diabetes. “I think and worry a lot,” she says. “I pray to the Buddha constantly.” So many people depend on her – if her health deteriorates further, she does not know who would take care of her husband, her granddaughters, and herself? What will happen if she gets even weaker than she is now? If she cannot work anymore? These are thoughts that she tries to control, she struggles to stop them from coming: “I feel depressed, but I must be determined. I must earn money, so that I can pay for my medicines. I’m determined to ‘live with the floods’ (‘*sống chung với lũ*’). I must try to stay within a range of around 7 or 8. If my blood sugar gets higher than that, I’m afraid of complications. I’m so worried. If I don’t manage to eat and exercise appropriately, this disease is very dangerous.” We ask with whom she shares these worries, and Bích replies that she confides in her neighbours. They are her age, and in the afternoon, when their grandchildren are playing outside, they often help each other looking after them. As for her son and daughter-in-law, Bích does not want to burden them. “I don’t want to put more on them. They have to think about their household economy, food for the children, clothes, medicines, diapers… they have so much to think about already.”

For many research participants, emotion work was an integral part of their daily lives with diabetes. Many had seen other villagers with diabetes develop frightening complications – ulcers, blindness, limb amputations – only to eventually die a premature death. They knew, therefore, only too well of the unsettling prospects that might lie ahead. In response, many deliberately adopted an optimistic attitude, trying to stave off fears by not thinking of what the future might bring. As one woman put it: “I tell myself to stay calm. One must live. I must overcome this and live. Thinking and speculating does not help. I don’t care. What will come will come.” Practically all research participants engaged in daily emotion work in this manner, trying to keep negative thoughts and feelings at bay. In some cases, people shared their concerns with their spouse – but many felt reluctant to confide in family members, fearing that this would place unnecessary emotional burdens on them. Outwardly, they therefore tried to adopt a positive and happy attitude, even in situations when they felt worried about emerging signs of complications or other significant problems. Participants’ everyday emotion work was, in this sense, double, consisting in active attention to their own emotions combined with safeguarding those of others, and particularly their loved ones, and constituting yet another aspect of everyday disease diplomacy.

## Discussion

This study shows that for people with type 2 diabetes in this rural area of Vietnam, diabetes self-care demands intense everyday work. Research participants balanced carefully between disease attentiveness and disease discretion, striving to attend to their disease while also downplaying it and preventing it from affecting others. Not only blood sugar balances, but also social balances, were vitally important to people. Across our sample, maintaining a positive social atmosphere – within and beyond the home – was of overarching importance.

The disease attentiveness exercised by research participants was, as the above cases illustrate, suffused by uncertainties – regarding how to manage blood sugars, which medicines to take, how to eat an appropriate diet, and how to handle negative emotions. As diabetes patients, they had often received minimal information and guidance from health care providers, and this lack of guidance generated doubt, anxiety, and uncertainty. These findings resonate with previous studies which have found large unmet needs for appropriate diabetes care and unclear diabetes management guidelines in low- and middle-income countries [26, 27]. A recent framework synthesis of chronic care models found that appropriate care for chronic conditions in LMIC requires a stronger focus on the quality of communication between health professionals and patients, more emphasis on essential medicines, and provision of diagnostics and trained personnel at decentralised levels of health care [28]. In the context of Vietnam, hospital overcrowding, lack of adequate primary level health care services, and lack of essential medicines have previously been found to place constraints on health care delivery [29, 30]. Although existing research indicates that motivations for appropriate disease management are high among people with chronic health conditions in Vietnam [31–34], lack of knowledge and limited health provider support often render self-care difficult. The comprehensive everyday life work that the present study documents must be seen on the background of these health systems constraints.

The disease discretion that research participants showed must be seen in the context of long-standing health beliefs in Vietnam. Previous research has shown that efforts to maintain balances – bodily as well as social – lie at the heart of everyday health practices in Vietnam [35, 36]. Sino-Vietnamese medical theories attribute ill health to inner imbalances caused by physical or emotional strain, or physical imbalances caused by external forces, such as wind, temperature, food, and drink [37]. Maintaining health, these theories hold, demands bodily and social harmony and stability. Optimism and positive thoughts are, therefore, considered to be health-enhancing, while negative thoughts may aggravate a given health problem. In northern Vietnam, when chronic disease strikes, people’s efforts to live well with it seem to draw on this existing fund of everyday health knowledge: the emphasis on socially smooth and discreet disease management found in the present study resonates with lay medical theories that emphasize positive and health enhancing states of mind.

Further, people’s striving to conceal their disease in order not to burden others must be seen on the background of local moral and spiritual beliefs. Ethnographic studies conducted across the globe have found chronic disease management to be strongly inflected by spirituality, as people find mental stamina in spiritual beliefs and in a sense of belonging to larger collectives [38, 39]. In Vietnam, such everyday spirituality draws on long-standing Confucian, Buddhist, and Taoist philosophies, together termed “the triple religion” [40]. In this moral cosmology, the universe is considered to consist of finely tuned socio-moral balances; a disturbance in one place will disturb and upset the entire system. This makes all living beings deeply interdependent, each individual holding responsibility for the well-being of all others. In this moral cosmology, self-sacrifice, compassion, and benevolence are prime moral virtues [41–43]. When people respond to a diabetes diagnosis through a recalibration of daily living habits, therefore, they do so with an awareness of the moral connotations of their habits and practices: the disease discretion and desire not to disturb or burden others that was so pronounced in this study must be seen in the context of these forms of local moral reasoning. In short, although diabetes is a relatively new disease to people in northern Vietnam, people’s daily self-care efforts are shaped by long-standing moral cosmologies, drawing on centuries of health beliefs and spiritual practices.

This study was not without limitations. Given that the participants were recruited through convenience sampling and that the sample was modest in size, the findings cannot be considered representative of people with diabetes in Vietnam. Validity, rather than representativity, was the main quality parameter for this ethnographic study [44]. We recommend, therefore, that similar research is carried out in other localities and among other groups of people in and beyond Vietnam, with a view to exploring the generalizability of the results.

## Conclusions

This study contributes to understanding how people in a rural area of Vietnam practice diabetes self-care, striving to integrate their diabetes into everyday lives. Our results show that even though motivations for appropriate disease management are strong, self-care can pose difficulties. Research participants balanced uneasily between disease attentiveness and disease discretion, trying to live up to biomedical requirements for disease management while also handling their diabetes in socially appropriate ways; that is, in ways that did not disturb or burden others. Daily diabetes work involved concerted efforts to maintain balances, bodily as well as social; to intervene and act, but to do so without upsetting the routine flows of daily lives. To capture this balancing act, we propose the term “everyday disease diplomacy.” People's inclination to practice diabetes self-care through careful disease diplomacy was driven by important social and moral concerns, but the research also points to risks that the price for such diplomacy can be less-than-optimal biomedical disease management – as when research participants opted to eat an ordinary diet in the attempt not to disturb their families; when they did not seek professional medical guidance regarding their medicines; or when they chose to keep emotional distress or concerns about emerging complications to themselves. These findings from Vietnam point to the importance of more sustained attention in research and health service provisioning to the implications of everyday disease diplomacy for NCD self-care.

## Data Availability

The datasets used and analysed during the current study are available from the corresponding author on reasonable request.

## Abbreviations

LMIC: Low- and Middle-Income Countries
NCD: Non-Communicable Disease
SDG: Sustainable Development Goal
T2D: Type 2 Diabetes
WHO: World Health Organization

## Footnotes

1 All authors contributed to data collection. The five Vietnamese researchers held responsibility for 5–6 cases each, while the Danish researcher took part in all 27 initial interviews with research participants. The team included two male and four female researchers of whom two hold PhDs (the first two authors), two are MDs (the third and last author), and four hold Public Health Master’s degrees (the fourth and fifth author). The five Vietnamese authors were employed at Thai Binh University of Medicine and Pharmacy and the Danish author at the University of Copenhagen at the time of the study. All authors have solid experience with ethnographic or public health field research in Vietnam.

2 Enrolment was divided into two different time periods for feasibility reasons. There were no differences between the two groups of participants. Relationships with participants were established when the study commenced. At recruitment, the researchers informed the participants of the background and motivation for the study and of the organizational set-up.
